# Astrocyte Clocks and Glucose Homeostasis

**DOI:** 10.3389/fendo.2021.662017

**Published:** 2021-03-18

**Authors:** Olga Barca-Mayo, Miguel López

**Affiliations:** ^1^ Circadian and Glial Biology Lab, Physiology Department, Molecular Medicine and Chronic Diseases Research Centre (CiMUS), University of Santiago de Compostela, Santiago de Compostela, Spain; ^2^ NeurObesity Lab, Physiology Department, Molecular Medicine and Chronic Diseases Research Centre (CiMUS), University of Santiago de Compostela, Santiago de Compostela, Spain

**Keywords:** astrocytes, circadian clock, metabolism, diabetes, glucose homeostasis

## Abstract

The endogenous timekeeping system evolved to anticipate the time of the day through the 24 hours cycle of the Earth’s rotation. In mammals, the circadian clock governs rhythmic physiological and behavioral processes, including the daily oscillation in glucose metabolism, food intake, energy expenditure, and whole-body insulin sensitivity. The results from a series of studies have demonstrated that environmental or genetic alterations of the circadian cycle in humans and rodents are strongly associated with metabolic diseases such as obesity and type 2 diabetes. Emerging evidence suggests that astrocyte clocks have a crucial role in regulating molecular, physiological, and behavioral circadian rhythms such as glucose metabolism and insulin sensitivity. Given the concurrent high prevalence of type 2 diabetes and circadian disruption, understanding the mechanisms underlying glucose homeostasis regulation by the circadian clock and its dysregulation may improve glycemic control. In this review, we summarize the current knowledge on the tight interconnection between the timekeeping system, glucose homeostasis, and insulin sensitivity. We focus specifically on the involvement of astrocyte clocks, at the organism, cellular, and molecular levels, in the regulation of glucose metabolism.

## Functional Hierarchy Of the Timekeeping System

**Figure 1:**
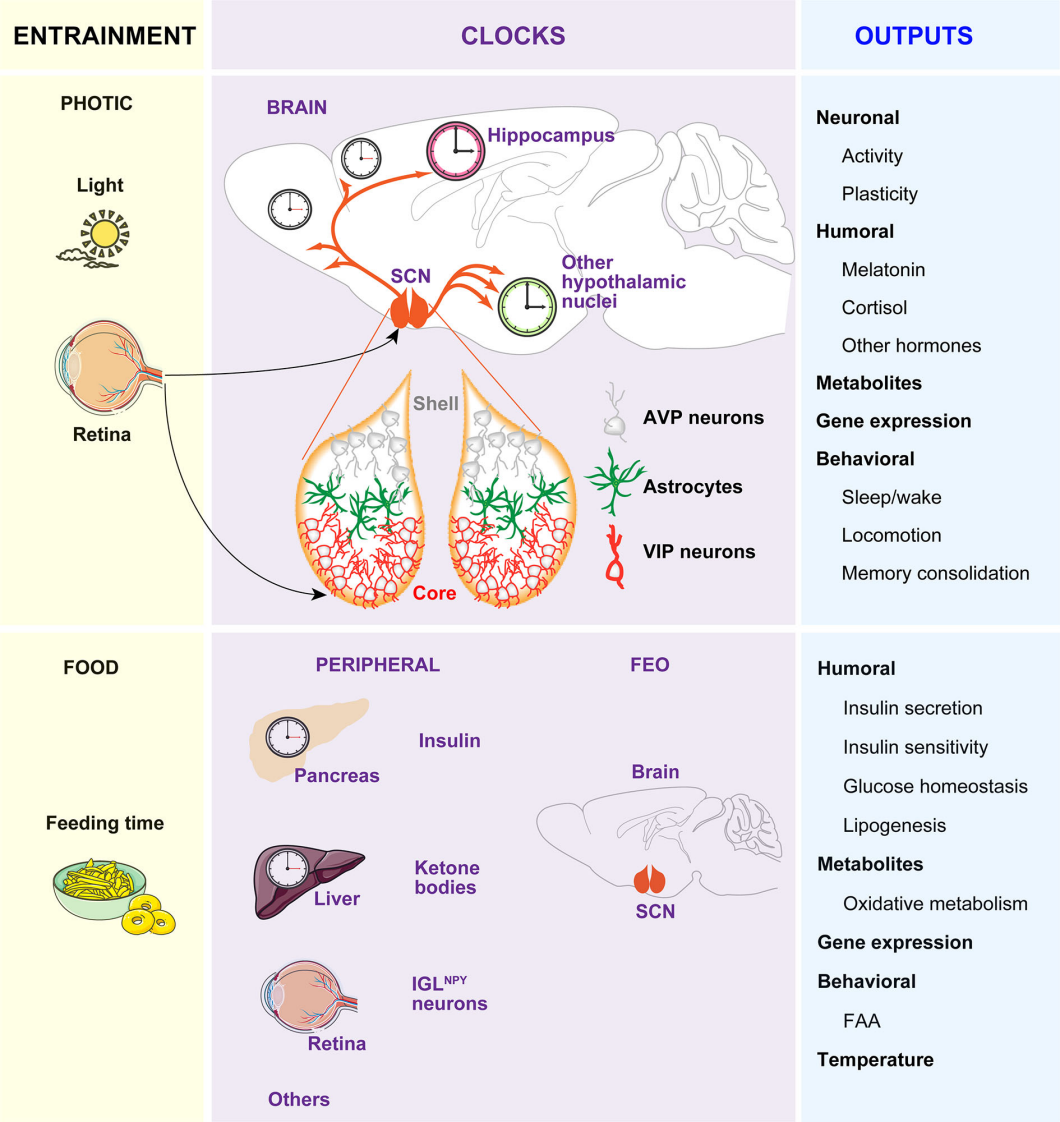
The circadian timing system. The timekeeping system is composed of two pacemakers (the SCN and the FEO), and peripheral clocks in other brain areas and peripheral tissues. Light inputs reaching the SCN *via* the retina and the retinohypothalamic tract, are the most important Zeitgeber for the SCN, which in turn synchronizes peripheral clocks through neural, endocrine, temperature, and behavioral signals. Feeding-related signals (INS and ketones bodies) generated by peripheral tissues and IGL^NPY^ neurons entrain the FEO, which regulate the outputs such as FAA.

The circadian (in Latin “circa”, around; “diem”, day) clock is an endogenous and self‐sustaining oscillator that operates with a periodicity of 24 hours (h) to maintain proper rhythms of the vast majority of physiological and behavioral processes, including food intake, energy balance, sleep-wake cycles and many others (1). In mammals, the timekeeping system comprises a pacemaker located in the hypothalamic suprachiasmatic nucleus (SCN) (2, 3), as well as non-SCN brain and peripheral clocks and cell-autonomous oscillators within virtually every cell type of the body (4, 5). In the absence of any time cue from the environment, these clocks free run with a period close to 24 h. To compensate discrepancies between this intrinsic period and the environmental cycle, circadian clocks entrain to external Zeitgebers (ZT, in German “time giver”). The light entrains the SCN to local time which in turn, conveys the temporal information to other clocks in the brain and peripheral tissues *via* neuronal, hormonal, or behavioral activity rhythms, such as the feeding-fasting and sleep-wake cycles, which serve as entrainment signals for extra-SCN clocks (6, 7) **(** **Figure 1** **)**.

While a substantial amount of information is known about the neuroanatomy of the SCN, the mechanisms of light-affected entrainment and the transmission of time cues to other brain areas or peripheral clocks are not yet fully understood. Briefly, the SCN is a heterogeneous and complex bilateral nucleus (8, 9) comprising approximately 10,000 self-oscillating neurons on each side, in mice (10–12). Each part is divided into two functional subgroups, one in the ventrolateral region and the other in the dorsal SCN. The ventrolateral region or core receives direct photic inputs from the intrinsically photosensitive retinal ganglion cells (ipRGCs) (13–15). Activation of the retino hypothalamic tract by light increases the firing of vasoactive intestinal polypeptide (VIP)-expressing SCN cells and VIP release. VIP neurons set and phase-shift the circadian time by VIPergic and γ-aminobutyric acid (GABA)ergic signaling to arginine vasopressin (AVP)-expressing neurons within the second subgroup of cells located in the dorsal SCN or shell **(** **Figure 1** **)**. Ultimately, this results in the induction of the so-called clock genes *Period1* (*Per1*) and *Period2* (*Per2*) (16) and subsequent time-of-day dependent phase responses of the SCN, thereby enabling entrainment to the light-dark (LD) cycle.

If food availability is restricted to a particular time of day or night (often referred to as time-restricted feeding, TRF), animals exhibit increased activity in anticipation of feeding (termed food anticipatory activity, FAA). Moreover, in this paradigm, the peripheral clocks shift their phase to preserve alignment with mealtime. The effects of TRF on peripheral clocks and/or behavior persist even when the feeding is out of phase with the LD cycle, and, indeed, can be uncoupled from the SCN, which remains synchronized to the light (17–19). Remarkably, in the absence of a functional SCN, FAA is preserved and food intake becomes an effective ZT capable of coordinating circadian rhythms of behavior, peripheral clock gene expression, and clock outputs, such as hormone secretion (20, 21). Thereby, the entrainment to TRF has been proposed to be independent of the SCN and driven by a food-entrainable oscillator (FEO) of still unknown location. A recent study reported that innervation of ipRGC to neuropeptide Y (NPY)-expressing intergeniculate leaflet (IGL) neurons in early postnatal stages, allow the entrainment to TRF in adults. In this model, TRF inhibitory signals from IGL^NPY^ neurons modulate the SCN activity, allowing the FEO signals to influence FAA (22).

As hypothalamic lesions and gene knockouts (KOs) targeting specific cell types in the hypothalamus, brainstem, or forebrain areas also impair FAA (23–29), it is reasonable to hypothesize that the FEO might be functionally distributed among brain areas that are competent to drive changes in behavior instead to be restricted to a particular brain region. In line with this idea, it was recently reported that the action of insulin (INS) and insulin-like growth factor-1 (IGF-1) triggered after feeding, on different brain regions, are necessary and sufficient for both the FAA and the phase-shift of body clocks (7). Remarkably, an elegant study showed that the signal that generates FAA might be synthesized in the liver. Specifically, it was shown that liver PER2 is required for hepatic-derived ketone bodies production which in turn signals the brain to induce FAA (30). In sum, the FEO may not be in a single tissue but it might be of systemic nature. In this context, the feeding-related signals, such as INS and ketones generated in peripheral tissues, entrain different brain regions competent to drive changes in behavior. Among these regions is included the SCN, which is tuned by innervation of ipRGCs to IGL during development to allow non-photic entrainment to food (22) **(** **Figure 1** **)**.

## Astrocyte Circadian Clocks

How the activity of a small number of SCN neurons is translated into rhythmic behaviors or physiology at the organism level? The human brain contains more than 100 billion cells, the majority being glial cells, coordinated by this endogenous clock to determine alertness waxes and wanes in a highly predictable manner over the course of a 24 h day (31). However, how this clock signaling is orchestrated within so many brain cells that lead to the cycle-to-cycle precision of circadian rhythmicity is unknown. Consequently, we face a lack of knowledge on the mechanisms by which circadian dysfunction affects a wide range of physiological processes such as metabolic imbalance, premature aging, and reduced longevity (32–38).

Astrocytes have long lived in the shadow of the neurons as they were thought to have mainly a structural role in the central nervous system. The recent findings showing the critical role of the astrocyte clock in the control of SCN function and circadian behavior (39–43) is a game-changing discovery that offers radically new research directions for therapy of brain diseases originated by environmental miss functioning of circadian rhythms or genetic factors affecting clock genes or outputs. This glial cell type is highly diverse in its morphological appearance, functional properties, and distribution among and within different brain regions (44, 45). However, they share three anatomical features that are crucial to understand its functional contributions to the timekeeping system.

Firstly, the longstanding concept that astrocytic processes interdigitate to create a scaffold for the neuronal organization, has been challenged by several studies showing that, *in vivo*, astrocytes are organized in nonoverlapping domains, i.e, with little interaction between adjacent cells (46–48). Thus, one astrocyte can coordinate the activity of multiple sets of contiguous synapses, *via* regulation of neurotransmitters levels in the synaptic cleft, *via* control of the extracellular space, or by releasing chemical signals that actively modulate synaptic transmission, often referred to as gliotransmitters. Specifically, astrocytes play an essential role in the coupling of SCN neurons by controlling both glutamate and GABA levels (40, 42, 43, 49–51). Moreover, SCN astrocytes undergo rhythmic structural rearrangements (52), along with rhythms in GFAP expression (53), which allows differential day/night coverage of VIP neurons to facilitate entrainment to light (52, 54). Similarly, in response to metabolic cues, astrocytes undergo structural and morphological changes to influence the synaptic inputs within the hypothalamic melanocortin system, which might ultimately affect the feeding behavior (55–58). Astrocytes also regulate the extracellular space (59–62), enabling the exchange of solutes between the cerebrospinal fluid and the interstitial space, a system referred to as glymphatic clearance. As the diffusion of SCN output signals is sufficient for rhythmic behavior (63, 64), daily changes in the glymphatic system may underly the synchronization among different brain regions across the circadian cycle. On the other hand, in *Drosophila*, a glial-released factor was shown to be critical for normal rhythmicity by regulating a neurotransmitter, pigment dispersing factor, acting on a receptor similar to that for VIP in mammals (65–67). Similarly, in rodents, astrocytes release gliotransmitters, such as ATP, in a circadian manner (68), and arrhythmic astrocytes alter VIP expression *in vivo* (40). However, whether ATP release and/or astrocytic rhythmic metabolism impact the activity patterns of VIP neurons is still unknown.

Secondly, astrocytes form a syncytium, *via* gap junctions, that allow the propagation of small signaling molecules through the glial network (69). Pharmacological inhibition of gap junctions in SCN slides (70, 71) and mouse models with deletion of the neuronal connexin-36, impairs the circadian pattern of neuronal activity without affecting the long-term synchronization of clock gene expression (72) and with mild effect on behavioral rhythms (73). Similarly, studies in mouse models with deletion of astrocytic specific connexins indicate that the astrocytic coupling in the SCN is dispensable for circadian rhythm generation and light-entrainment (74). However, recently, a long-range function of astrocytes for the transmission of timing cues to distant neural populations was investigated *in vitro* with microfluidic devices that allowed compartmentalizing distinct neuronal populations connected through a network of astrocytes. In this paradigm, astrocytes were able to synchronize the clock of segregated cortical neuronal populations if intercellular communication between the glial network and/or calcium signaling were intact (75). Whether astrocytes are involved in the spatial transmission of timing cues in other extra-SCN brain regions *in vivo* is still unknown.

Thirdly, the exchange of metabolites and hormones through the blood-brain barrier (BBB) relies on astrocytes and is dependent both on the sleep/wake state and in the circadian clock (59, 76–80). As hypothalamic astrocytes have a crucial role in sensing nutrients such as glucose and fatty acids (56, 81–83) and express receptors for leptin (84, 85), IGF-1 (86), thyroid hormone (87), INS (57), and glucocorticoids (GCs) (88, 89) among others, they could link or coordinate peripheral and central oscillators. For example, astrocytes, as well‐known targets of GCs, might be sensitive to the negative feedback loop of the hypothalamus‐pituitary‐adrenal axis. It is widely accepted that GC signaling can reset peripheral clocks but not the central pacemaker because SCN neurons do not express the GC receptor (90). However, astrocytic feedback loops, *via* GC signaling, could explain the so far puzzling results showing that the Per1‐Luc phases of SCN were affected significantly when adrenalectomized animals were treated with hydrocortisone (6).

## Molecular Dynamics of the Clock

The Nobel Prize in Physiology or Medicine in 2017 was awarded to three Chronobiologists who first cloned the Droshopila *Period* gene in 1984 (91, 92). This finding allowed us to understand how the timekeeping system anticipates the environmental changes related to the Earth’s rotation in most, if not all, living organisms.

**Figure 2:**
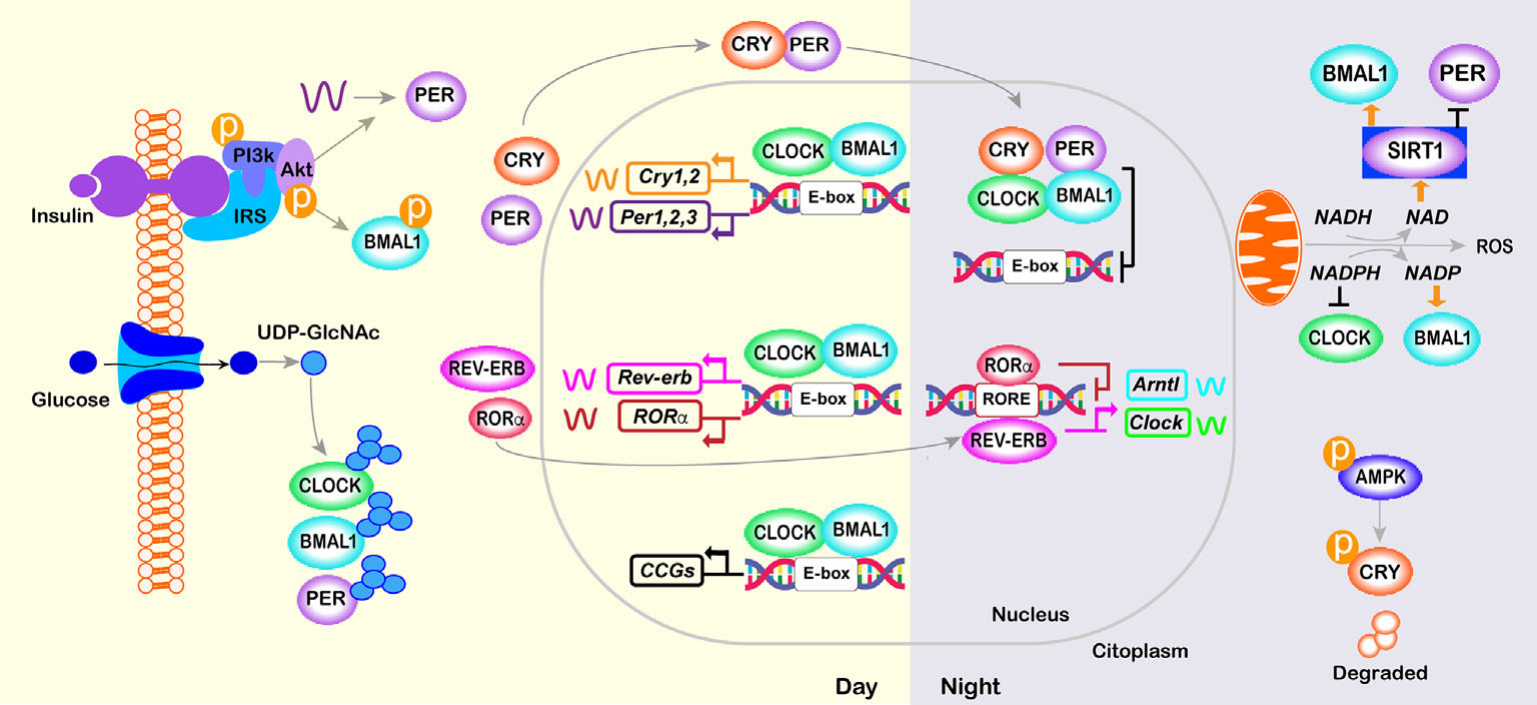
Core molecular clock network. The mammalian molecular clock consists of a transcriptional-translational feedback loop involving the clock proteins CLOCK, ARNTL, PER, and CRY and the nuclear receptors REV-ERB and ROR. The positive limb (CLOCK and BMAL1) heterodimerizes and activates the transcription of downstream genes, including *Per, Cry, Rorα* and *Rev-erb*. The negative limb proteins (PERs and CRYs) multimerize and inhibit CLOCK/BMAL1. In a secondary loop, *Bmal1* and *Clock* are regulated by the repressor REV-ERB and its opposing nuclear receptor RORα, which bind competitively to the shared element RORE, thus repressing or activating the transcription of the *Bmal1* or *Clock* gene, respectively. Post-translational modifications of core-clock factors can also regulate transcription (e.g., deacetylation of BMAL1 or PER2 by SIRT1, BMAL1 phosphorylation or PER2 translation by INS signaling; O- GlcNAcylation of CLOCK, BMAL1 and PER2; and CRY1 phosphorylation by AMPK).

The molecular clock involves rhythmic and self‐sustained transcriptional–translational feedback loops (TTFLs) of clock genes/proteins **(** **Figure 2** **)**. The E‐box specific transcription factors BMAL1 (Brain and muscle Arnt‐like protein‐1) and CLOCK (Circadian locomotor output cycles kaput) are the positive limb of the TTFL, which heterodimerize to activate transcription of the repressors *Per1*/2/3 and *Cryptochrome* genes (*Cry1*/2) (93, 94). The negative loop comprises PER/CRY heterocomplex that, upon accumulation, lead to the degradation of BMAL1/CLOCK dimers, thus inhibiting their own transcription (95). Hence, a new cycle of PER and CRY protein accumulation begins, generating rhythmic changes in the levels of the core clock transcripts and proteins that persist for approximately 24 h (96) **(** **Figure 2** **)**. In a secondary feedback loop, the CLOCK- BMAL1 complex controls the rhythmic expression of the genes encoding the REV-ERB nuclear hormone receptors and ROR (97). In turn, REV-ERB and ROR compete for the same RORE elements within the *Clock* and *Bmal1* promoter, repressing or activating, respectively, *Clock* and *Bmal1* transcription **(** **Figure 2** **)**.

Direct targets of CLOCK/BMAL1, referred to as clock-controlled genes (CCGs), include genes that are critically involved in rhythmic processes such as feeding behavior, sleep-wake cycle, and glucose homeostasis (34, 98) **(** **Figure 2** **)**. In turn, metabolic state sensing pathways also alter the molecular clock in anticipation of the LD cycle. Specifically, during feeding, anabolic processes are triggered by the activation of the INS-AKT-mTOR pathway, whereas during fasting, AMP-activated protein kinase (AMPK) activation triggers catabolic processes and inhibits mTOR activity (99). BMAL1 phosphorylation and PER2 translation are regulated by the INS-AKT-mTOR pathway that is activated in the postprandial state (7, 100–102). Similarly, in peripheral tissues, AMPK1, which senses cellular ATP levels, modulate CRY1 phosphorylation and thus its rhythmic degradation (103). Additionally, high levels of glucose control the period length *via* O- GlcNAcylation of CLOCK, BMAL1, and PER2 (104–106). The molecular clocks are also sensitive to the ratio of reduced to oxidized nicotinamide adenine dinucleotide (NAD) and flavin adenine dinucleotide (FAD), which are indirect sensors of cellular energy status. Oxidation of NAD is under control of the clock and, in turn, prevents the deacetylation and this the transcriptional activity of CLOCK-BMAL1 complex by Sirtuin 1 (SIRT1) and poly-ADP-ribosylation mediated by poly(ADP-ribose) polymerase 1 (107–110) **(** **Figure 2** **)**.

In summary, the circadian system ensures a temporal partitioning of catabolic and anabolic reactions synchronizing organism metabolism to the feeding-fasting cycle. However, as the connection of metabolism and the circadian clock works in both directions (111) is not surprising that animal models of genetic clock defects display metabolic alterations and that clock alterations can be found in metabolically challenged conditions (112).

## Coordination of Glucose Homeostasis by Central and Peripheral Clocks

Glucose homeostasis is optimal when fasting-feeding and rest-activity cycles, hormonal rhythms, and central and peripheral clocks oscillate in synchrony with each other to ensure that the timing cues and tissue responsiveness are achieved at the right time. During the active phase, metabolic tissues such as the liver, muscle, and fat are very sensitive to INS to guarantee that glucose uptake is properly achieved after food intake. Conversely, these tissues are more resistant to the hormone during the fasting phase, to facilitate the endogenous glucose production and free fatty acid (FFA) secretion (113–115). In this section, we discuss the clocks in the tissues and organs involved in the control of glucose homeostasis and describe their role in the regulation of INS sensitivity and secretion.

**Figure 3:**
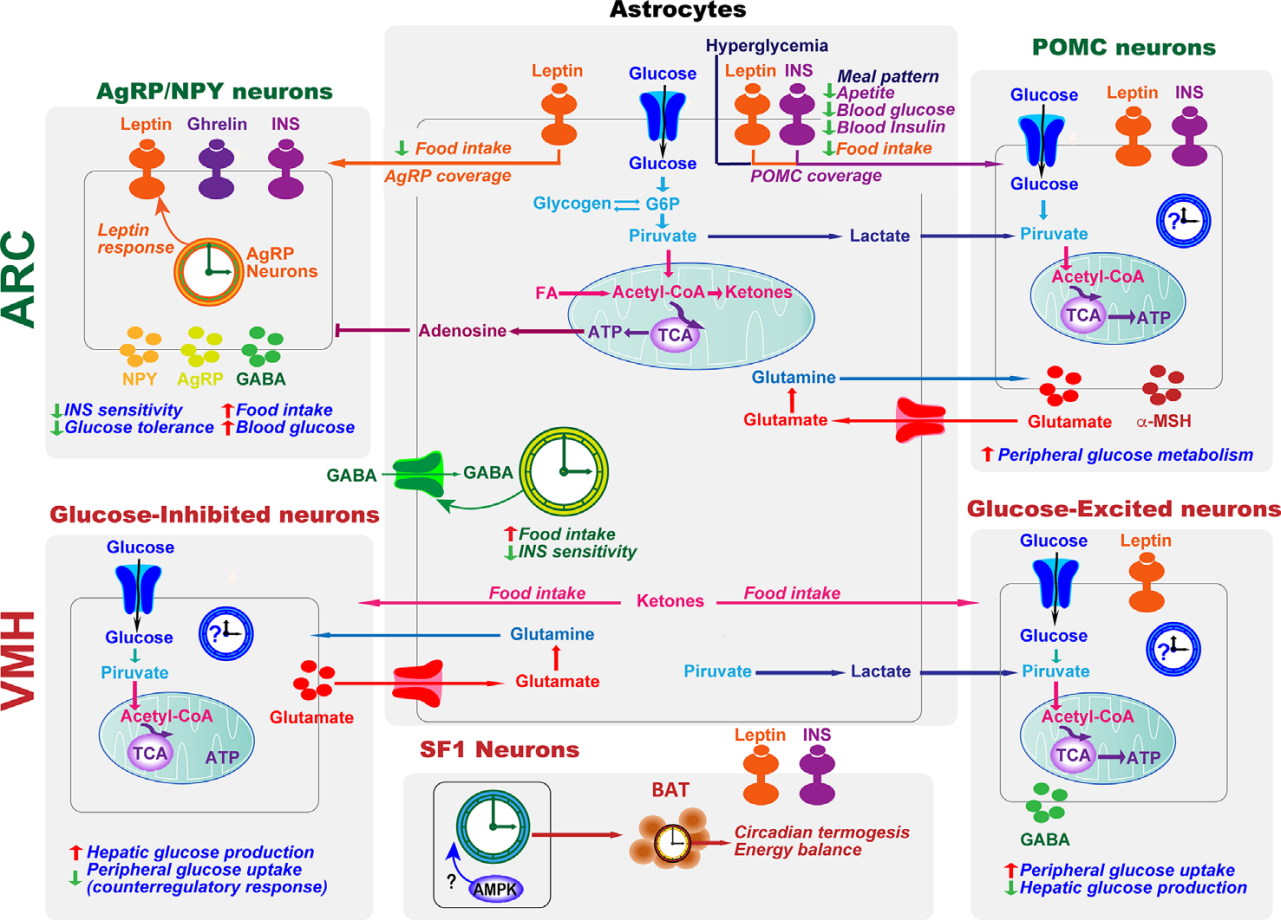
Astrocytes and clocks modulate hypothalamic glucose-sensing mechanisms. The ability of POMC and NPY/AgRP neuronal populations in ARC to alter energy metabolism is due to their sensitivity to several circulating signals, including hormones, such as leptin and insulin (INS), and nutrients. Hypothalamic astrocytes provide neurons with structural support and nutrients. Moreover, hyperglycemia, INS, and leptin signaling in this glial cell type lead to changes in the astrocytic coverage of POMC and/or AgRP neurons to regulate glucose sensing. Glucose transported into astrocytes can be metabolized to lactate, which is released and taken up by neurons and metabolized into pyruvate to serve as a glycolytic substrate. Astrocytes can also modulate synaptic transmission by uptake of neurotransmitters from the synaptic cleft (glutamate and GABA) and by releasing gliotransmitters such as adenosine, which inhibits AgRP neurons. In the ARC, the astrocyte circadian clock might control food intake and glucose homeostasis by regulating the uptake of GABA. On the other hand, the clock in AgRP neurons is required for coordinating leptin response and glucose metabolism. VMH neurons include glucose-sensing cells, referred to as glucose-excited and glucose-inhibited neurons. The activation of glucose-excited neurons leads to decreased hepatic glucose production and increased peripheral glucose uptake. VMH glucose-inhibited neurons are activated in response to hypoglycemia. In recurrent hypoglycemia, high accumulation of lactate enhances the glucose-excited neuronal activity and consequent GABA release, inhibiting the counterregulatory response. A high-fat diet increases astrocyte ketone bodies production, which are exported to VMH neurons to ultimately control food intake. Subsets of VMH neurons also express SF1. These SF1 neurons contain a clock that modulates energy expenditure by regulating cyclic thermogenesis in brown adipose tissue (BAT). As hypothalamic AMPK modulates BAT thermogenesis (116), and has a crucial role in the molecular clock, it would be interesting to investigate its involvement in the control of rhythmic BAT thermogenesis by the SF1 neuronal clock. TCA, tricarboxylic acid cycle.

The hypothalamus integrates glucose-sensing mechanisms with multiple effector pathways to precisely coordinate hepatic glucose production, muscle and fat glucose uptake, and endocrine pancreas function **(** **Figure 3** **).** Briefly, in the arcuate nucleus of the hypothalamus (ARC), orexigenic agouti-related peptide (AgRP)-producing neurons, and anorexigenic neurons releasing the pro-opiomelanocortin (POMC)-derived peptide, α-melanocyte-stimulating hormone, together with the neurons expressing the melanocortin 4 receptor (MC4R), are essential for glucose sensing (117). AgRP neurons are glucose-inhibited cells whereas POMC neurons are glucose-excited (118). In general terms, competitive binding of α-MSH and AgRP to MC4Rs defines the activation magnitude of downstream pathways and effectors. Furthermore, POMC and AgRP neurons project to numerous extrahypothalamic and hypothalamic regions, including the ventromedial nucleus (VMH) (119), which is crucial to initiate the glucose counter-regulatory response to hypoglycemia (120) **(** **Figure 3** **)**. On the other hand, hypothalamic astrocytes respond to hyperglycemia by retraction of the coverage around POMC neurons to modify meal patterns (56). Consistently, astrocytes sense INS and leptin to co‐regulate behavioral responses and metabolic processes *via* the control of brain glucose uptake and the glial ensheathment of POMC neurons, respectively (57, 58). Moreover, deletion of leptin receptors in astrocytes reduces the physiological anorexigenic response to this hormone and enhances fasting or ghrelin-induced hyperphagia (58). Additionally, stimulation of astrocytes with ghrelin modify glutamate and glucose metabolism as well as glycogen storage by decreasing GLUT2, glutamine synthetase, and lactate dehydrogenase, and increasing glutamate uptake, glycogen phosphorylase, and lactate transporters, which might modulate the signals/nutrients reaching neighboring neurons (121). Finally, activated astrocytes release adenosine to inhibit AgRP neurons, thus suppressing the ghrelin-mediated increase of food intake (122, 123) **(** **Figure 3** **)**.

As the SCN imposes the sleep-wake cycle and food intake occurs in the active period, the involvement of the pacemaker in controlling glucose homeostasis and systemic INS sensitivity is indirect (124). Indeed, BMAL1 deletion in SCN does not affect the body weight despite complete loss of rhythmic behavior (20). Moreover, the circadian locomotor activity but not the metabolic disturbances of *Bmal1−/−* mice were rescued by restoring BMAL1 expression in the SCN (125). Similarly, mice with astrocyte‐specific deletion of BMAL1 show altered energy balance and glucose homeostasis despite their circadian locomotor activity is not lost (39–43). Thus, peripheral and/or extra-SCN hypothalamic clocks, but not the SCN, might have a crucial role in developing glucose intolerance and INS resistance. In line with this idea, BMAL1 ablation within SF1 neurons in the VMH is sufficient to alter energy expenditure (126). Additionally, AgRP-specific ablation of BMAL1 increases hepatic gluconeogenesis (127). However, currently, little is known about the specific physiological functions of extra SCN brain clocks. This knowledge could be highly valuable for biomedical understanding and future therapeutic advancement in the metabolic imbalance associated with circadian disruption.

Metabolic tissues involved in glucose homeostasis also have autonomous clocks that govern and adjust their daily metabolic function or outputs. For example, in the liver, with an essential role as a buffer for glucose variations arising from rhythmic food consumption, ablation of the local clock leads to hypoglycemia restricted to the fasting phase and exaggerated glucose clearance (128). Moreover, it was reported that while hepatic glycogenesis is controlled by CLOCK (129), gluconeogenesis in the fasted state, is under the regulation of the repressor CRY1 (130–133). On the other hand, efficient glucose uptake by the hepatocytes at the beginning of the active phase depends on the rhythmic expression of glucose transporters and glucagon receptor (134, 135). Another tissue with a key role in the control of glucose homeostasis is the skeletal muscle. This tissue is responsible for 70-80% of INS-stimulated glucose uptake in the postprandial state (136). Interestingly, INS sensibility in muscle is controlled both by light and the local clock (137–140). Specifically, it was shown that photic inputs entrain diurnal changes in clock gene expression and INS sensitivity in muscle *via* SIRT1 in SF1 neurons (140). On the other hand, deletion of the autonomous clock in muscle is sufficient to cause local INS resistance (137). Glucose uptake is also dependent on rhythmic INS action in the white adipose tissue (WAT) (141). However, contrary to liver or skeletal muscle, ablation of the local clock in WAT do not impact glucose homeostasis and INS sensitivity (142), suggesting that lipid mobilization is mainly regulated by the hypothalamic actions of INS and leptin. Finally, brown adipose tissue (BAT), which relays in FFA and glucose supply to regulate thermogenesis, is highly flexible in terms of glucose uptake potential and can significantly contribute to whole-body glucose metabolism under some conditions. In this tissue, INS-stimulated glucose uptake is regulated by the VMH and AgRP neurons (143–145), as well as by the local clock (146, 147). Interestingly, mouse and human BAT express a red-light-sensitive protein, OPN3 (148), which increases glucose uptake upon red light stimulation. Recently, an elegant study demonstrated that animals reared without violet light show increased responses to β-agonists, which in humans activate BAT, lower blood glucose levels, and increase and INS sensitivity. This effect was mediated by a violet light-sensing photoreceptor Opsin 5 (OPN5) in glutamatergic warm-sensing hypothalamic preoptic area neurons (149). Altogether, these studies open the possibility of modulating glucose homeostasis by manipulating environmental light.

Altogether this suggests that perturbed rhythms of the central and/or tissue clocks might lead to a mismatch between hepatic glucose production, muscle glucose uptake, and carbohydrate intake which could contribute to elevated levels of glucose and an imbalance between lipid storage in WAT and lipid oxidation in the brown adipose tissue. Furthermore, hyperglycemia in diabetes is traditionally attributed to reduced INS sensitivity in skeletal muscle and liver but also coupled to decreased INS secretion by the pancreas. Not only glucose homeostasis and INS sensitivity is under control of the local clocks in most of the above-mentioned metabolic tissues, but the pancreatic clock also controls rhythmic INS secretion (150, 151). Indeed, ablation of the pancreatic clock, in mice, is sufficient to cause hypoinsulinemia and hyperglycemia (150, 152–154).

Interestingly, feeding-related hormones involved in the control of glucose homeostasis are timing cues for circadian behaviors. For example, leptin is involved in the regulation of sleep-wake cycles (155, 156); ghrelin, stimulate FAA in mice (157) and INS action, triggered after feeding, is a critical entrainment signal for the FEO (7). Thus, an intriguing unsolved question concerns how the neurocircuits involved in glucose homeostasis and the central or peripheral clocks crosstalk and coordinate appropriate metabolic and/or circadian responses.

## Integration of Glucose Homeostasis by Astrocyte Clocks and Cellular Metabolism

As brain metabolic pathways are compartmentalized between astrocytes and neurons, the coordination of both cell types is needed to meet the high energy requirements of synaptic transmission and correct brain function (158). Thereby, it is not surprising that hypothalamic glucose sensing requires an intact metabolic coupling between astrocytes and neurons (159). Interestingly, a big percentage of components of cellular metabolic pathways are direct targets of the molecular clock (96, 111, 160). Together, this suggests that the close association between altered glucose homeostasis and circadian disruption may arise from a shared defect in the astrocyte-neuron metabolic coupling. In turn, this might impact the neurocircuitry governing energy and glucose homeostasis or alter metabolic adaptations to hypoglycemia in the diabetic brain. In this section, we discuss the current evidence that supports this hypothesis.

The major energy source for the brain is glucose, which is taken up by astrocytes and neurons *via* glucose transporters (GLUTs) (161–163). In the hypothalamus, the neuron-astrocyte glucose coupling expands beyond the accomplishment of energy requirements. For instance, astrocytes actively cooperate with hypothalamic neurons in detecting circulating glucose levels and in the generation of proper systemic metabolic responses (164). Not only astrocytic GLUT2 activity is involved in the regulation of systemic glucose homeostasis in rodents (165–167), but restoring astrocytic GLUT2 reestablish the counterregulatory response to low-glucose in GLUT2 deficient mice (165). Remarkably, rhythmic GLUTs expression (137, 168, 169) is impaired in the brain of experimental streptozotocin-induced diabetes rats (169). Moreover, 24 h oscillation in glucose levels may modulate the expression of clock genes and transcriptional outputs within hypothalamic neurons involved in glucose homeostasis (170). Whether astrocyte-neuron metabolism in hypothalamic glucose sensing and associated-systemic response in the normal or diabetic brain relies on the astrocyte molecular clock remains to be investigated. However, this idea is reinforced by the recent finding that deletion of *Bmal1* in astrocytes impairs INS sensitivity and glucose homeostasis (39).

Further, glucose can be stored as glycogen or metabolized in the glycolytic pathway to produce pyruvate, which is either transferred into mitochondria or converted to lactate. According to the “Astrocyte-to-Neuron Lactate Shuttle” (ANLS) hypothesis (171), lactate is primarily produced by astrocytes and transferred to neurons, where it is converted to pyruvate for aerobic energy production in mitochondria (**Figure 3**). Thereby, the production and the release of lactate by astrocytes is directly linked to neuronal activity, as showed in orexin neurons (172, 173). Consistent with the ANLS hypothesis, brain lactate levels increase during the awake state when neuronal firing rates are higher and vice versa, leading to a 24 h rhythm of lactate concentration (174, 175). In turn, astrocytic lactate release regulates the sleep-wake cycle (74) and entrain forebrain oscillators between states of alertness and tiredness by controlling the DNA binding of CLOCK/BMAL1 (176). Remarkably, during hypoglycemia in diabetes patients, brain lactate levels drop while its infusion increased brain lactate levels compared to healthy subjects (177–179). These findings suggest increased lactate use as a metabolic substrate, impaired astrocyte lactate release, or perturbed compensatory metabolic mechanisms in the diabetic brain. Whether these effects underlie a potential astrocytic dysrhythmia is currently unknown.

More than half of the energy used by neurons during fasting derives from ketones bodies (180) synthesized by astrocytes. The astrocytic switch from glucose to FFA utilization (181, 182) to produce ketones is particularly enhanced in the hypothalamus (181), where stimulate neuropeptides critically involved in glucose sensing and energy homeostasis (83, 182, 183) (**Figure 3**). On the other hand, recurrent exposure to low glucose, mimicking variations often seen in patients with diabetes, results in increased astrocytic ketogenesis (184–186), likely to preserve brain ATP production (187). In turn, increased astrocytic ketogenesis alters INS signaling and consequently glucose homeostasis (183). Despite it was shown that *Per2* controls the hepatic production of ketone bodies (30), whether hypothalamic ketogenesis is under the control of the astrocyte clock remains to be investigated. Similarly, whether arrhythmic astrocytes impair glucose homeostasis by contributing to the increased ketogenesis is unknown and could be crucial for therapeutic interventions involving the potentiation of the astrocyte clock.

On the other hand, glucose, as well as glutamine, can be metabolized to uridine diphosphate N-acetylglucosamine (UDP-GlcNAc) through the hexosamine biosynthetic pathway. The reversible enzymatic post-translational modification of proteins (on serine and threonine residues) with UDP-GlcNAc as glucose donor is termed O-GlcNAcylation. This process is conserved across species as occurs both in mouse brains and Drosophila neurons. While in conditions of glucose hypometabolism, brain levels of O-GlcNAc-modified proteins are reduced (188), hyperglycemia increases GlcNAcylation of proteins related to the INS pathway, thus contributing to INS resistance (189). Hyperinsulinemia is also associated with increased GlcNAcylation (189) of proteins involved in the pathology of diabetes, such as glycogen synthase, a major gatekeeper of glucose metabolism (190, 191). On the other hand, O-GlcNAcylation serves as a metabolic sensor to control the circadian period length *via* modification, and thus changes in the transcriptional activity of CLOCK and PER2 (104). As neurons depend on astroglial glucose and glutamine, this suggests that O-GlcNAcylation of the neuronal clocks might be coupled to astrocyte metabolism. However, further studies are needed to verify this hypothesis.

The metabolic endpoint of glycolysis and the mitochondrial metabolism is ATP generation, which apart from being used to fuel biological reactions is released to the extracellular space (eATP) (eATP) (192). Remarkably, INS stimulates ATP release from astrocytes (193). In turn, eATP leads to rapid upregulation of glycolysis (194) and promotes glucose uptake into both neurons and astrocytes (195). eATP is also a signaling molecule that acts on purinergic, ionotropic P2X, and G-protein coupled P2Y receptors to regulate neuronal activity (196). In the hypothalamus, NPY and AgRP neurons express P2X2R (197), whereas SF-1 neurons are excited by ATP *via* the P2X4 receptor (198). eATP released by astrocytes can also be metabolized to adenosine. While activation of the A1 receptor by adenosine inhibits appetite-stimulating AgRP neurons (122) **(** **Figure 3** **)**, in astrocytes modulate sleep homeostasis (46). Thus, the circadian release of astrocytic ATP (199) and the circadian activity of enzymes involved in adenosine synthesis (200) suggest a central role of the astrocyte clock in modulating both processes. On the other hand, decreased ATP production activates the AMPK pathway to impact the circadian clock *via* degradation of CRY1 (103). Further investigation will clarify whether astrocytic rhythmic ATP release entrains the neuronal clocks *via* circadian activation of AMPK to control energy homeostasis and circadian sleep-wake changes in the brain.

Glutamate, the major excitatory neurotransmitter in the adult CNS, is released from neurons and recycled by astrocytes to form glutamine **(** **Figure 3** **),** which is returned to neurons and used as a precursor for synthesizing glutamate and GABA (201). The uptake of glutamate by astrocytes, critical for neuronal activity (202), is metabolically expensive and requires an increase in glycolysis and lactate production (171). Remarkably, control of glutamate and GABA levels, coupled to astrocyte rhythms (40–43, 203), is necessary for the generation of molecular and behavioral rhythms and, is also critically involved in the modulation of hypothalamic neural circuits controlling glucose homeostasis (39, 204, 205). However, excessive demands on astrocytes, in response to a decrease in glucose levels, impair glutamate uptake (206), altering the glutamatergic signaling to delay the onset of the normal counterregulatory response to hypoglycemia (206). Conversely, intake of an obesogenic diet rapidly increases hypothalamic glutamatergic signaling (207) and the expression of astrocytic glutamate transporters (208). It is reasonable to hypothesize that chronic elevated glutamatergic signaling, associated with diet-induced obesity, increases the metabolic demands on astrocytes to prevent glutamate-induced excitotoxicity. This in turn negatively impacts their ability to support neuronal activity thus, contributing to hypothalamic synaptic dysfunction and the death of POMC neurons (55, 209, 210). Altogether, this suggests that the regulation of glutamate and GABA levels might be a key astrocyte circadian function in normal physiology and likely involved in the alterations of the diabetic brain.

The enteric nervous system is gaining more attention in the last few years. While most of the research focused on the enteric neurons, less attention was directed towards the enteric glial cells (EGCs). Glucose enters the body *via* the gastrointestinal (GI) tract, and conversely, diabetes-induced GI dysfunction is related to increased apoptosis of EGC in the myenteric plexus (211). On the other hand, the gut clock synchronized by food intake (27, 212) regulates the expression of brush border disaccharidases and glucose absorption to the habitual feeding period (168). Moreover, Glucagon-like peptide-1secretion by enteroendocrine L-cells, with an important role in regulating glucose homeostasis (213, 214), is under control of the clock (215). In the gut, also anatomical and metabolome patterns of the microbiota undergo rhythmic fluctuations, resulting in system-wide effects on host circadian transcriptional, epigenetic, and metabolite cycles (216). Interestingly, repeated jet lag in mice disturbs the intestinal microbiome leading to reduced glucose tolerance (216). Similarly, fecal transfer from jet-lagged humans into germ-free mice impaired glucose tolerance (216). These findings suggest that the microbiome clock has an important role in the development of INS resistance due to repeated phase shifts. Altogether, this indicates that the contribution of the gut clock, specifically in the enteric glia, to the control of glucose homeostasis warrants further work.

Altogether, this data indicates that further investigations about the role of the astrocyte clock in maintaining the cycle-to-cycle precision of cellular metabolism and neural rhythmic behavior could be crucial to counteract the systemic metabolic abnormalities associated with circadian disruption.

## Circadian Disruption and Diabetes

In this section, we review the current evidence about the contribution of genetic or environmental factors (such as exposure to artificial light-dark cycles, disturbed sleep, shift work, and jet lag) that impact the timekeeping system to the development of insulin resistance and type 2 diabetes.

In humans, mutations in several clock genes are strongly associated with obesity, INS resistance, and type 2 diabetes. Specifically, it was reported associations between single nucleotide polymorphisms in *ARNT* and T2DM  (217), specific haplotypes of *CLOCK* and obesity (218, 219), and between polymorphisms in *CRY2* and elevated fasting glucose (220, 221). In line with the human clock gene mutation studies, rodent models with genetic deletions of core-clock genes (in either a whole-body or a tissue-specific manner) showed INS resistance, obesity, and type 2 diabetes (32, 125, 142, 222, 223). Remarkably, deletion of *Bmal1* in astrocytes in mice is sufficient to phenocopy the obesity, INS resistance, and glucose intolerance of *Bmal1-/-* constitutive KO mice (39), suggesting that robust astrocyte circadian rhythms could preserve whole-body homeostasis and metabolic health.

The central pacemaker anticipates and synchronizes the daily function of peripheral tissues according to the entrainment by natural changes in light. With the advent of affordable artificial lighting and the 24/7 lifestyle of our society, humans began to experience increased exposure to artificial lights and irregular light schedules. These environmental changes lead to a desynchronization between the internal clock and the external ZT, a phenomenon referred to as circadian misalignment. Remarkably, human and animal studies have linked obesity and type 2 diabetes with increased light exposure during naturally dark hours (224–230). In turn, exposure to bright morning light increases fasting and postprandial glucose levels in patients with type 2 diabetes (230). Despite its relevance for health, the molecular and cellular mechanisms of normal and pathological phototransduction in the SCN are unclear. For instance, VIP rhythm, with a key role in synchronizing SCN neurons to each other and with the LD cycle (231, 232), is driven by the LD cycle and not by the circadian clock (233). Thereby, the mechanism by which deletion of *Bmal1* in astrocytes constantly elevates VIP levels (40) remains unknown. Indeed, constant illumination increases VIP levels lengthening the circadian period and resulting in two or more peaks in daily activity (234), a circadian locomotor pattern that resembles that of mice with arrhythmic astrocytes (40). Consistently, studies in *Drosophila* showed that glial-specific genetic manipulations lead to circadian arrhythmicity due to alterations on a clock neuron peptide transmitter (pigment dispersing factor) that acts on a receptor similar to that for VIP in mammals (65, 66). These studies suggest that the astrocyte clock might facilitate the entrainment to light and therefore, to the light-induced phase shifts in physiology and behavior. Further investigations on the mechanism underlying circadian entrainment to light are critical for understanding why aberrant light exposure, disrupts circadian physiology leading to diabetes and INS resistance.

Evidence from epidemiological and experimental studies indicate that sleep restriction or disturbance, increases the risk of obesity and type 2 diabetes (235–239) likely due to increased food intake (237, 238), altered sympathovagal balance (240, 241), and increased circulating levels of catecholamines (242) or cortisol (241, 242). Interestingly, astrocytes modulate mammalian sleep homeostasis by controlling adenosine A1 receptors (243). The circadian release of the astrocytic transmitter ATP (199) as well as the circadian activity of enzymes involved in the synthesis of adenosine in areas of the brain related to sleep (200) suggests a central role of astrocytes in modulating circadian sleep-wake changes in the brain. Further studies will be needed to understand the importance of astrocyte clocks in the relationship between circadian sleep disorders and diabetes.

Whereas light is the dominant timing cue for the SCN, the time of meals represents the main ZT for peripheral clocks. Therefore is not surprising that extended/erratic eating patterns, such as in shift workers or subjects under experimental circadian misalignment, showed decreased glucose tolerance and insulin sensitivity (244–250). Indeed, a short-term circadian misalignment protocol of 8 days in humans is sufficient to cause higher blood glucose and insulin levels (249). It is reasonable to hypothesize that disturbance of nutrient fluxes or the misalignment of central and peripheral clock rhythms might contribute to the pathophysiology of insulin resistance at the tissue level. For instance, a mismatch between hepatic glucose production, muscle glucose uptake, and carbohydrate intake could contribute to elevated glucose levels, while an imbalance between lipid storage in WAT, lipid oxidation in BAT, and hepatic lipid production might contribute to ectopic lipid accumulation. However, to improve or prevent the metabolic alterations caused by circadian misalignment we need to further understand the mechanisms involved in the entrainment of both central and peripheral circadian clocks. Remarkably, as astrocytes are at the interface between vessels and neurons, they are in a privileged position to act as metabolic sensors of systemic cues that entrain the peripheral clocks, such as GCs, INS, or IGF1 (7, 40, 203). Further studies will clarify whether those metabolic cues might play a crucial role in communicating time-of-feeding to the astrocyte molecular clock linking the periphery and the CNS clocks.

## Conclusion

A large body of evidence from human or animal studies demonstrated the circadian regulation of glucose homeostasis and INS sensitivity. However, the exact mechanisms involved in the metabolic derangements resulting from circadian disruption are not fully understood. Emerging groundbreaking findings, showing that astrocytes are pivotal for the circadian regulation of behavior and whole-body energy and glucose homeostasis, could provide a new cellular target to tune physiological responses operating on different timescales according to metabolic status. A key question that remains to be investigated is how the astrocyte clock is entrained to lead to the cycle-to-cycle precision of circadian rhythmicity in the SCN and/or in extra SCN clocks. Therefore, we face a lack of knowledge on the mechanisms by which astrocyte circadian dysfunction affects such a wide range of physiological processes. Understanding these mechanisms will be a challenge for years to come but a crucial aspect in designing better therapies, such as clock agonists, for diabetes. With this knowledge, the use of chronotherapies or temporally directed therapeutics to improve human metabolic health* *will be a matter of time.

## Author Contribution

All authors contributed to the article and approved the submitted version. OB-M conceptualized and wrote the manuscript and made the figures. OB-M and ML discussed and edited the manuscript and the figures.

## Conflict of Interest

The authors declare that the research was conducted in the absence of any commercial or financial relationships that could be construed as a potential conflict of interest.
